# Feasibility of dynamic risk assessment for patients with repeated trans-arterial chemoembolization for hepatocellular carcinoma

**DOI:** 10.1186/s12885-019-5495-6

**Published:** 2019-04-16

**Authors:** Yehyun Park, Beom Kyung Kim, Jun Yong Park, Do Young Kim, Sang Hoon Ahn, Kwang-Hyub Han, Jong Eun Yeon, Kwan Soo Byun, Hye Soo Kim, Ji Hoon Kim, Seung Up Kim

**Affiliations:** 1 Department of Internal Medicine, Institute of Gastroenterology, Yonsei University College of Medicine, 50 Yonsei-ro, Seodaemun–gu, Seoul, 120–752 South Korea; 2 Liver Cirrhosis Clinical Research Center, Seoul, South Korea; 3 Division of Gastroenterology and Hepatology, Department of Internal Medicine, Korea University College of Medicine, 148, Gurodong-ro, Guro-gu, Seoul, 08308 South Korea

**Keywords:** Hepatocellular carcinoma, Trans-arterial chemoembolization, Risk prediction, Hepatoma arterial-embolization prognostic score

## Abstract

**Background:**

Hepatoma arterial-embolization prognostic (HAP) score and its modifications (modified HAP [mHAP] and mHAP-II), consisting of some or all of the following factors of tumor size, number, alpha-fetoprotein, bilirubin, and serum albumin, have been found to predict outcomes after trans-arterial chemoembolization (TACE) for hepatocellular carcinoma (HCC). We investigated the feasibility of using HAP-related risk scores for dynamic risk assessment during repeated TACE.

**Methods:**

A total of 619 HCC patients treated with TACE from two institutions between 2003 and 2010 were included.

**Results:**

Patients with A-B class risk scores showed significantly better survival than those with C-D class risk scores at the first (median 43.7 vs. 21.5 months for mHAP-II, 35.2 vs. 10.2 months for mHAP, and 39.8 vs. 18.6 months for HAP; all *P* < 0.001) and the second rounds of TACE (38.6 vs. 17.2 months for mHAP-II, 30.0 vs. 8.5 months for mHAP, and 32.6 vs. 17.3 months for HAP; all *P* < 0.001). Sequential assessment of risk scores at the second TACE round was applied for patients with A-B class risk scores at the first TACE round, which further identified two subgroups of A-B and C-D class risk scores with different outcomes (median survival 40.6 vs. 19.6 months for mHAP-II, 31.2 vs. 16.9 months for mHAP, and 35.8 vs. 21.0 months for HAP; all *P* < 0.001). Compared with mHAP and HAP, mHAP-II showed the highest likelihood ratio (22.61 vs. 14.67 and 13.97, respectively), highest linear trend (24.43 vs. 19.67 and 14.19, respectively), and lowest Akaike information criteria value (1432.51 vs. 3412.29 and 2296.98, respectively).

**Conclusions:**

All HAP-related risk scores dynamically predicted outcomes during repeated TACE. Sequential risk assessment using mHAP-II best identified optimal candidates for repeated TACE.

**Electronic supplementary material:**

The online version of this article (10.1186/s12885-019-5495-6) contains supplementary material, which is available to authorized users.

## Background

Despite the availability of curative treatment modalities, such as liver transplantation, surgical resection, and radiofrequency ablation, the outcomes of patients with hepatocellular carcinoma (HCC) remain poor. This is because most HCC patients are not suitable candidates for these curative treatments, due to their advanced disease stage and poor liver function at the time of diagnosis [1, 2]. Accordingly, non-curative treatments, such as trans-arterial chemoembolization (TACE), radioembolization, and sorafenib, are used in patients with advanced HCC [3–5].

Based on the survival advantages of TACE, compared to best supportive care, reported in randomized trials and a subsequent systematic review [6–8], international guidelines have recommended TACE for patients with HCC of Barcelona Clinic Liver Cancer (BCLC) intermediate stage (B) or those with early stage disease who are not candidates for percutaneous ablation, liver resection, or transplantation [9]. However, differences in survival have been reported among a series of patients treated with TACE [10], probably because of the heterogeneity of liver function and the tumor burden among patients of the same disease stage. Thus, as proposed in previous studies [11, 12], it is important to select candidates who will benefit from TACE.

Hepatoma arterial-embolization prognostic (HAP) score, which consists of four tumor-related variables (alpha-fetoprotein [AFP] level and tumor size) and liver function-related variables (serum albumin and total bilirubin levels), has been proposed for predicting outcomes after TACE [13]. In addition, two modifications of HAP score have been proposed: modified HAP score (mHAP), which excludes total bilirubin from the HAP score [14], and mHAP-II score, in which tumor number is a constituent variable [15].

The above HAP-related risk scores have been shown to provide acceptable accuracy in risk assessment of patients with HCC treated with TACE. However, because HAP-related risk scores comprise values determined at the time of the initial TACE and because remnant tumor burden and liver function can change after each TACE session, their dynamic prognostic performance needs to be confirmed. Thus, we evaluated the feasibility of dynamic risk assessment using HAP-related risk scores during repeated TACE rounds in patients with HCC and compared their prognostic performance.

## Methods

### Patient eligibility

Consecutive treatment-naïve patients diagnosed with HCC treated with TACE as a first-line therapy from 2003 to 2009 (Liver Center, Severance Hospital, Yonsei University College of Medicine) and 2003 to 2010 (Liver Center, Guro Hospital, Korea University of College of Medicine) were included in this retrospective multicenter cohort study.

The exclusion criteria were 1) inadequate target lesion with infiltrative pattern, non-arterial enhancement, or the largest lesion less than 1 cm; 2) presence of primary malignancy in another organ; 3) tumor invasion to the main portal vein or presence of extrahepatic tumor lesions; 4) Child-Pugh class C; 5) BCLC stage D, 6) presence of uncontrolled functional or metabolic diseases, and 7) TACE as a bridge to transplantation (Additional file 1: Figure S1).

The study protocol was designed in accordance with the ethics guidelines of the 1975 Declaration of Helsinki, and the study was approved by the institutional review boards of Severance Hospital and Korea University Guro Hospital. Written informed consent was not acquired because this study was a retrospective study.

### Diagnosis and staging of HCC

HCC diagnosis was made based on the guidelines proposed by the Korea Liver Cancer Study Group [16]. Tumor staging was assessed using the BCLC staging system [17].

### TACE procedure and follow-up

Detailed information on the TACE procedure has been described in a previous study [15]. Briefly, after angiography of the superior mesenteric and hepatic arteries, conventional TACE was performed by selective infusion of a mixture of 5 mL of iodized oil contrast medium (lipiodol; Guerbet) and either 50 mg of adriamycin or cisplatin at 2 mg/kg body weight, followed by embolization using gelatin sponge particles with a diameter of 1 mm (Cutanplast; Mascia Bruneili, S.p.a.). Super-selective embolization was performed using a 2.0-Fr microcatheter (Progreat alpha; Terumo). Embolization was performed until stasis was achieved.

A contrast-enhanced CT or MRI of the liver was performed at 4 to 6 weeks after TACE to assess the effect of embolization on the tumor. The radiologic response to TACE was based on the modified Response Evaluation Criteria in Solid Tumors (mRECIST) on CT or MRI [3, 4]. In patients with a residual arterially enhancing viable tumor, TACE was repeated at 6- to 8-week intervals, if clinically indicated. In patients with complete tumor necrosis, a contrast-enhanced CT or MRI was repeated every 3 to 6 months [3, 4]. In patients who were not candidates for repeated TACE, alternative treatment was performed at the physician’s discretion. The TACE procedures and follow-up protocols were largely the same between two institutions, and there was no major change in TACE practice during the study period.

### Study design

After pooling patient data from the two institutions, the study population was divided into a group with favorable expected outcomes (A-B class for three risk scores) and one with unfavorable expected outcomes (C-D class for three risk scores) at each TACE. The detailed scoring systems of HAP-related risk scores are summarized in Additional file 2: Table S1 [13–15]. Then, since we previously showed that mHAP-II score, as well as HAP and mHAP scores, predicted survival outcomes for treatment-naïve patients with HCC treated with TACE, we validated the prognostic performance of HAP-related risk scores during repeated TACE (A-B vs. C-D class HAP-related risk scores at the first and second TACE rounds). Next, we investigated whether the sequential use of HAP-related risk scores during repeated TACE held any prognostic value (A-B class risk score at the first TACE→A-B class risk score at the second TACE vs. A-B class risk score at the first TACE→C-D class risk score at the second TACE vs. C-D class risk scores at the first TACE). Measurement of tumor sizes for HAP-related scores after repeated TACE was based on mRECIST [3, 4]. Finally, we investigated the most appropriate risk score with which to determine subgroups with different prognoses when the sequential use of risk scores was applied during repeated TACE (Additional file 3: Figure S2).

### Statistical analysis

Baseline patient and tumor characteristics are presented as the median (interquartile range; IQR) or n (%), as appropriate. The Mann-Whitney test and Fisher’s exact test were used to compare characteristics between the two institutes, as appropriate. Survival was defined as the time from the date of each TACE until the date of death or last follow up. Survival curves were plotted using the Kaplan-Meier method, and median survival times with their 95% confidence intervals (CIs) are reported. The log-rank test was used to compare the survival difference between the groups.

Variables including components of HAP-related risk scores were evaluated using univariate and multivariate Cox regression analyses to identify predictive factors for survival. Variables with *P* < 0.05 in the univariate analysis were included as candidate variables in the multivariate Cox regression analysis to identify independent predictors of survival in the respective TACE sessions. The adjusted hazard ratios (HRs) and 95% Cis for variables were also calculated. The prognostic performance of HAP-related risk scores at the first and the second rounds of TACE was assessed using areas under receiver-operating curves (AUROCs) to predict mortality at 1 to 5 years of follow up. A comparison between AUROCs was made using the DeLong test.

To compare the homogeneity and discriminatory ability of HAP-related risk scores, the likelihood ratio test and the linear trend test were used. Furthermore, Akaike information criteria (AIC) were calculated to demonstrate which HAP-related risk score was more explanatory and informative for risk assessment of survival (a smaller AIC indicates the preferred risk score).

All values of *P* < 0.05 were considered to indicate statistical significance. Data were analyzed using SPSS 20.0 for Windows (SPSS Inc., Chicago, IL) and MedCalc Software (version 12.7.2, MedCalc Software bvba, Ostend, Belgium).

## Results

### Baseline characteristics

A total of 677 treatment-naïve patients with HCC treated with TACE as the first-line therapy were considered eligible (297 from Severance Hospital and 380 from Guro Hospital). After excluding 58 patients according to our exclusion criteria, 619 patients were included for statistical analysis (275 from Severance Hospital and 344 from Guro Hospital) (Additional file 1: Figure S1 and Additional file 2: Table S2).

The baseline characteristics and liver-related biochemical tests of the study population at the first and second TACE rounds are shown in Table 1. At the first TACE round, the median age of the study population (489 men and 130 women) was 59 years. The majority of HCCs were related to hepatitis B virus (HBV) infection (*n* = 422, 68.2%). The majority of patients had well-preserved liver function with a Child-Pugh class of A (*n* = 516, 83.4%). The median diameter of the largest measurable lesion was 3.5 cm, and 285 (46.0%) patients had multifocal HCC lesions. Segmental portal vein invasion was identified in 74 (12.0%) patients.

**Table 1:** Patient characteristics

Variables	At the first TACE (*n* = 619)	At the second TACE (n = 514)
Age (years)	59 (52–66)	–
Male gender	489 (79.0)	–
Etiology		
HBV/ HCV/ others	422 (68.2)/ 90 (14.5)/ 107 (17.3)	–
Child-Pugh class		
A/ B	516 (83.4)/ 103 (16.6)	436 (84.8)/ 78 (15.2)
BCLC stage		
0/ A/ B/ C	36 (5.8)/ 261 (42.2)/ 227 (36.7)/ 95 (15.3)	128 (24.9)/ 174 (33.9)/ 121 (23.5)/ 91 (17.7)
Tumor size (cm)	3.5 (2.1–6.5)	2.1 (1.3–4.0)
Tumor number		
Unifocal/ multifocal	334 (54.0)/ 285 (46.0)	451 (87.7)/ 63 (12.3)
Alpha-fetoprotein (ng/mL)		
≤400/ > 400	471 (76.1)/ 148 (23.9)	458 (89.1)/ 56 (10.9)
Segmental portal vein invasion	74 (12.0)	74 (14.4)
Total bilirubin (mg/dL)	0.9 (0.6–1.3)	1.0 (0.6–1.3)
Serum albumin (g/dL)	3.9 (3.4–4.2)	3.7 (3.3–4.1)

Variables are expressed as medians (interquartile range) or n (%)

*TACE* trans-arterial chemoembolization, *HBV* hepatitis B virus, *HCV* hepatitis C virus, *BCLC* Barcelona Clinic Liver Cancer

Of the study population, 514 (83%) patients underwent additional TACE, and their characteristics at the second TACE are shown in Table 1. Among these 514 patients who underwent a second round of TACE, 396 did so because of a residual lesion or incomplete response to the first TACE, whereas the others (*n* = 118) received second TACE on an on-demand basis to treat recurred HCC. The median interval between the first and second TACE rounds among these patients was 43 days (range, 16–90 days).

### Follow up and survival outcomes of the study population

By the end of the follow-up period, 457 of 619 (73.8%) patients had died (185 from Severance Hospital and 272 from Guro Hospital). The median survival of the study population was 30.0 (95% CI 26.8–33.2) months. The survival rates at 1, 3, and 5 years after the first TACE round was 76.7, 44.2, and 27.3%, respectively (Additional file 4: Figure S3).

### Independent risk factors for mortality at the first and second TACE

In univariate analysis, male gender (HR 1.29), Child-Pugh class B (HR 1.56), BCLC stage B-C (HR 1.64), the five components of mHAP-II score (HR 1.98 for tumor size > 7 cm; HR 1.88 for tumor number ≥ 2; HR 1.62 for AFP > 400 ng/mL; HR 1.29 for total bilirubin > 0.9 mg/dL; and HR 1.51 for serum albumin < 3.6 g/dL), and C-D class mHAP-II score (HR 2.07) were significant risk factors associated with mortality at the first TACE (all *P* < 0.05) (Additional file 2: Table S3). At the second TACE, all of the above risk factors, except male gender (*P* = 0.461) and total bilirubin (*P* = 0.066), also significantly predicted mortality (Additional file 2: Table S3).

Upon multivariate analysis of the significant variables in the univariate analysis, without BCLC stage and Child-Pugh class to avoid potential bias due to multi-collinearity, the five components of mHAP-II, including tumor size > 7 cm (HR = 1.90), tumor number ≥ 2 (HR = 1.73), AFP > 400 ng/mL (HR = 1.58), total bilirubin > 0.9 mg/dL (HR = 1.31), and serum albumin < 3.6 g/dL (HR = 1.45), at the first TACE independently predicted mortality (all *P* < 0.05) (Table 2). Similarly, all constituent variables of mHAP-II score, except for total bilirubin (*P* = 0.241), at the second TACE independently predicted mortality (Table 2).

**Table 2:** Independent predictors of survival from a multivariate Cox proportional hazards model at the first and second rounds of TACE

Variables	At the first TACE		At the second TACE	
	HR (95% CI)	*P* value	HR (95% CI)	*P* value
Male gender	1.09 (0.86–1.38)	0.501	–	–
Tumor size (cm)		< 0.001		< 0.001
≤7	1		1	
>7	1.90 (1.53–2.36)		1.91 (1.36–2.69)	
Tumor number		< 0.001		< 0.001
Unifocal	1		1	
Multifocal	1.73 (1.43–2.09)		1.78 (1.45–2.19)	
Alpha-fetoprotein (ng/mL)		< 0.001		< 0.001
≤400	1		1	
>400	1.58 (1.27–1.95)		2.31 (1.63–3.28)	
Total bilirubin (mg/dL)		0.006		0.241
≤0.9	1		1	
>0.9	1.31 (1.08–1.58)		1.14 (0.91–1.43)	
Serum albumin (g/dL)		< 0.001		0.004
≥3.6	1		1	
<3.6	1.45 (1.19–1.77)		1.39 (1.11–1.75)	

TACE; transarterial chemoembolization; HR, hazard ratio; CI, confidence interval

### Survival outcomes according to A-B vs. C-D class risk scores at the first and second TACE

Since C-D class risk scores significantly predicted mortality in the univariate analysis and since the five components of HAP-related risk scores were selected as independent risk factors for mortality, we investigated survival outcomes according to A-B vs. C-D class risk scores at the first and second TACE.

**Fig. 1:**
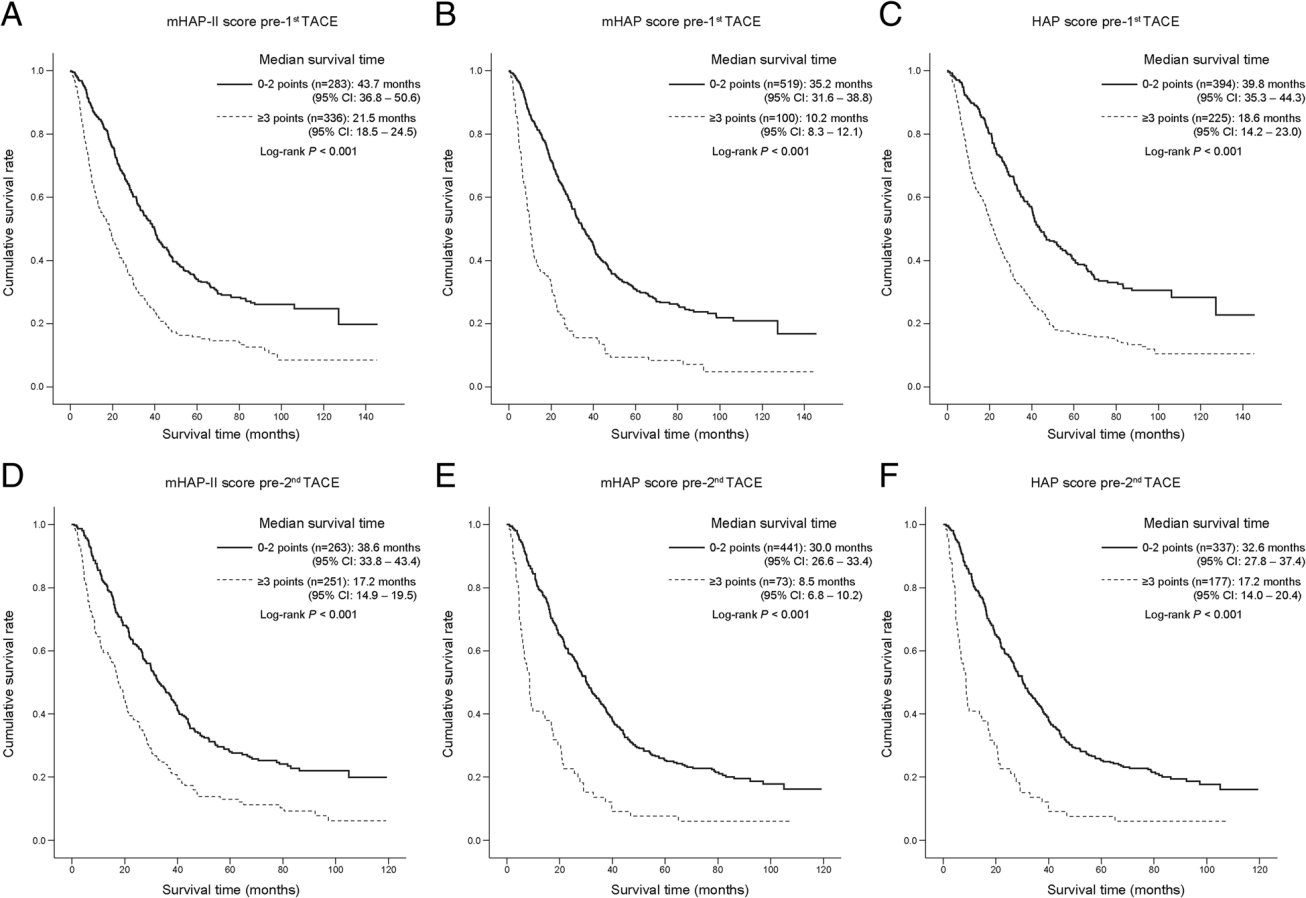
Kaplan-Meier survival curves of A-B vs. C-D class mHAP-II, mHAP, and HAP risk scores at the first (**a**-**c**) and second (**d**-**f**) rounds of TACE. Patients with A-B class risk scores showed significantly better survival than those with C-D class risk scores at the first and second TACE rounds. mHAP, modified hepatoma arterial-embolization prognostic; TACE, trans-arterial chemoembolization

At the first TACE (*n* = 619), patients with A-B class risk scores showed significantly better median survival than those with C-D class risk scores (43.7 [*n* = 283, 46%] vs. 21.5 months [*n* = 336, 46%] for mHAP-II; 35.2 [*n* = 519, 84%] vs. 10.2 months [*n* = 100, 16%] for mHAP; 39.8 [*n* = 394, 64%] vs. 18.6 months [*n* = 225, 36%] for HAP; all *P* < 0.001) (Fig. 1a-c, Table 3, Additional file 3: Figure S2). The significant survival differences according to A-B vs. C-D class risk scores were also maintained at the second TACE (*n* = 514) (all *P* < 0.001) (Fig. 1d-f, Table 3, Additional file 3: Figure S2). The survival rate at 1, 3, and 5 years after the first and second rounds of TACE according to risk scores are summarized in Table 3. The HRs for mortality for C-D class risk scores were 2.01–2.73 at the first TACE and 1.97–2.64 at the second TACE (Table 3).

**Table 3:** Survival outcomes according to risk scores at the first and second rounds of TACE

Risk scores	Median survival (95% CI)	Survival rate			Cox regression	
		1-year	3-years	5-years	HR (95% CI)	*P* value*
At the first TACE (n = 619)						
mHAP-II						
A-B (*n* = 283)	43.7 (36.8–50.6)	89.8%	59.7%	40.1%	1	
C-D (*n* = 336)	21.5 (18.5–24.5)	65.7%	31.3%	16.8%	2.07 (1.71–2.50)	<0.001
mHAP						
A-B (*n* = 519)	35.2 (31.6–38.8)	83.6%	49.7%	30.8%	1	
C-D (*n* = 100)	10.2 (8.3–12.1)	40.2%	15.5%	9.3%	2.73 (2.17–3.45)	<0.001
HAP						
A-B (*n* = 394)	39.8 (35.3–44.3)	86.1%	53.9%	34.1%	1	
C-D (*n* = 225)	18.6 (14.2–23.0)	60.2%	27.4%	15.7%	2.01 (1.66–2.42)	<0.001
At the second TACE (n = 514)						
mHAP-II						
A-B (*n* = 263)	38.6 (33.8–43.4)	86.5%	51.9%	33.3%	1	
C-D (*n* = 251)	17.2 (14.9–19.5)	60.3%	24.2%	11.2%	2.28 (1.86–2.80)	<0.001
mHAP						
A-B (*n* = 441)	30.0 (26.6–33.4)	78.9%	42.6%	25.0%	1	
C-D (*n* = 73)	8.5 (6.8–10.2)	39.4%	12.7%	7.0%	2.64 (2.02–3.44)	<0.001
HAP						
A-B (*n* = 337)	32.6 (27.8–37.4)	81.6%	46.5%	27.8%	1	
C-D (*n* = 177)	17.2 (14.0–20.4)	57.5%	22.8%	12.3%	1.97 (1.60–2.41)	<0.001

*P* value* indicates a comparison with A-B class of risk scores

*HAP* hepatoma arterial-embolization prognostic, *mHAP* modified HAP, *CI* confidence interval, *TACE* trans-arterial chemoembolization

### Dynamic risk assessment using risk scores during repeated TACE

**Fig. 2:**
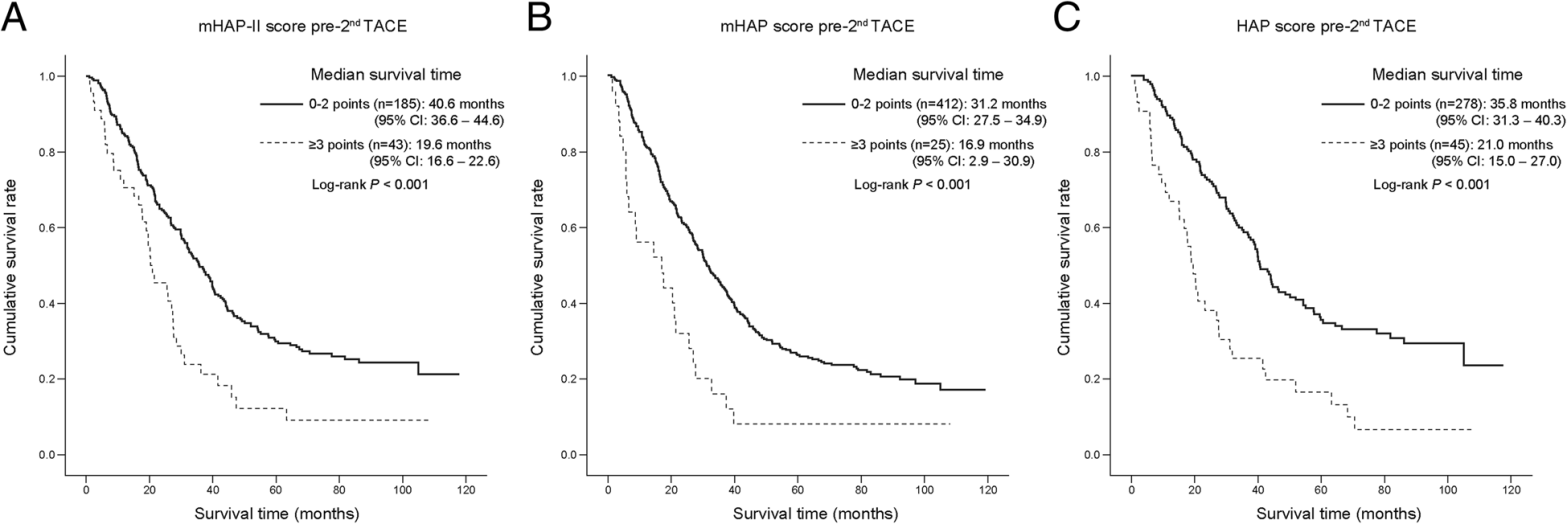
Kaplan-Meier survival curves of A-B vs. C-D class mHAP-II (**a**), mHAP (**b**), and HAP (**c**) risk scores at the second TACE among patients who showed A-B class risk scores at the first TACE. Patients with A-B class risk scores showed significantly better survival than those with C-D class risk scores at the second TACE. mHAP, modified hepatoma arterial-embolization prognostic; TACE, trans-arterial chemoembolization

Among the 283 patients with mHAP-II A-B risk scores at the first TACE, 228 patients underwent a second TACE in an on-demand manner. Of these, 185 (81%) and 43 (19%) patients showed mHAP-II A-B risk scores and mHAP-II C-D risk scores, respectively, at the second TACE (Additional file 3: Figure S2). The median survival of patients with mHAP-II A-B risk scores at the second TACE was significantly better than that of patients with mHAP-II C-D risk scores (40.6 vs. 19.6 months; *P* < 0.001) (Fig. 2a, Table 4**,** Additional file 3: Figure S2). Similar findings were also observed when mHAP and HAP were applied (Fig. 2b-c, Table 4**,** Additional file 3: Figure S2). The survival rates at 1, 3, and 5 years for the A-B and C-D class risk scores at the second round of TACE among patients who were categorized with A-B class risk at the first TACE round are summarized in Table 4.

**Table 4:** Survival outcomes according to the sequential use of risk scores during repeated TACE rounds

Risk scores	Median survival (95% CI)	Survival rate			Cox regression	
		1-year	3-years	5-years	HR (95% CI)	*P* value*
mHAP-II (n = 619)						
A-B at the first TACE (n = 283)						
No second TACE (*n* = 55)	–	–	–	–	–	–
A-B at the second TACE (*n* = 185)	40.6 (36.6–44.6)	89.6%	58.6%	35.5%	1	–
C-D at the second TACE (*n* = 43)	19.6 (16.6–22.6)	66.8%	25.4%	16.4%	2.15 (1.48–3.12)	<0.001
C-D at the first TACE (*n* = 336)	21.5 (18.5–24.5)	65.7%	31.3%	16.8%	2.31 (1.85–2.87)	<0.001
mHAP (n = 619)						
A-B at the first TACE (n = 519)						
No second TACE (*n* = 82)	–	–	–	–	–	–
A-B at the second TACE (*n* = 412)	31.2 (27.5–34.9)	81.2%	44.6%	26.2%	1	
C-D at the second TACE (*n* = 25)	16.9 (2.9–30.9)	56.0%	16.0%	8.0%	2.28 (1.49–3.48)	<0.001
C-D at the first TACE (*n* = 100)	10.2 (8.3–12.1)	40.2%	15.5%	9.3%	2.91 (2.29–3.69)	<0.001
HAP (*n* = 619)						
A-B at the first TACE (n = 394)						
No second TACE (*n* = 71)	–	–	–	–	–	–
A-B at the second TACE (*n* = 278)	35.8 (31.3–40.3)	84.6%	49.6%	30.0%	1	
C-D at the second TACE (*n* = 45)	21.0 (15.0–27.0)	70.6%	23.9%	12.1%	1.84 (1.30–2.62)	0.001
C-D at the first TACE (*n* = 225)	48.6 (14.2–23.0)	60.2%	27.4%	15.7%	2.17 (1.77–2.66)	<0.001

*P* value* indicate the comparison with A-B of risk scores at the first TACE

*TACE* trans-arterial chemoembolization, *CI* confidence interval, *HR* hazard ratio, *mHAP* modified hepatoma arterial-embolization prognostic

When the study population was stratified into three groups according to risk stratification at the first and second rounds of TACE, as previously described (A-B class risk score at the first TACE → A-B class risk score at the second TACE vs. A-B class risk score at the first TACE → C-D class risk score at the second TACE vs. C-D class risk score at the first TACE), patients with C-D class risk scores at the first TACE showed the highest risk of mortality (HR 2.17–2.91) and those with A-B class risk scores at the first TACE, but C–D class risk scores at the second TACE showed intermediate risk (HR 1.84–2.28) when compared with patients with A-B class risk scores at both the first and second TACE rounds (Table 4).

### Prognostic accuracy of the sequential use of risk scores during repeated TACE

Among the studied risk scores, mHAP-II, compared with mHAP and HAP, showed the highest homogeneity (likelihood ratio, 22.61 vs. 14.67 and 13.97, respectively), highest discriminatory ability (linear trend, 24.43 vs. 19.67 and 14.19, respectively), and lowest AIC value (1432.51 vs. 3412.29 and 2296.98, respectively), indicating that mHAP-II shows the best prognostic performance in dynamic risk assessment during repeated rounds of TACE (Table 5).

**Table 5:** Prognostic accuracy of the sequential use of risk scores during repeated TACE rounds to predict mortality

Risk scores	Likelihood ratio (χ^2^)	Linear trend (χ^2^)	AIC
mHAP-II	22.61	24.43	1432.53
mHAP	14.67	19.67	3412.29
HAP	13.97	14.19	2296.98

The model with a higher χ2 value by the likelihood ratio test and the linear trend test was considered the better model for homogeneity and discriminatory ability. Furthermore, lower values for Akaike information criteria were considered indicative of better discriminatory ability

*AIC* Akaike information criteria, *mHAP* modified hepatic arterial-embolization prognostic

## Discussion

**Fig. 3:**
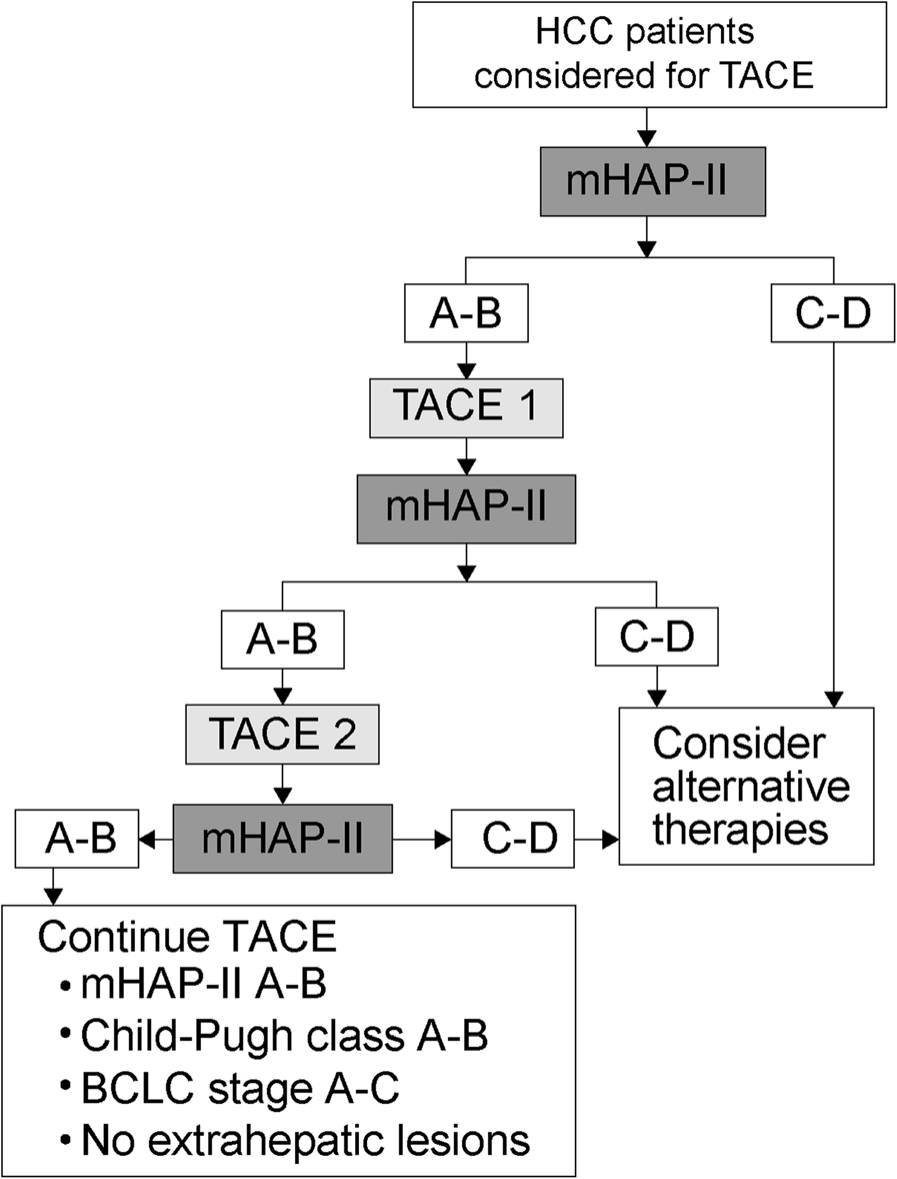
A proposed management strategy based on the sequential use of mHAP-II scores to select optimal candidates for TACE

In this multicenter study, we validated the prognostic value of three HAP-related risk scores (HAP, mHAP, and mHAP-II) before a first and second round of TACE in our pooled patient population. The results demonstrated the feasibility of dynamic risk assessment by sequential evaluation of HAP-related risk scores over repeated TACE sessions. mHAP-II score exhibited the greatest prognostic accuracy in terms of homogeneity and discriminatory ability. Based on these results, we propose an integrated algorithm that includes sequential evaluation of mHAP-II score to identify optimal candidates for TACE as the first-line treatment modality and to determine the benefits of subsequent TACE sessions. This algorithm may also facilitate the identification of subgroups of patients at risk of early treatment failure over repeated TACE sessions (Fig. 3).

Our study has several strengths. First, the sample size was large (*n* > 600), which enabled the assessment of the prognostic performance of HAP-related risk scores after sequential TACE sessions. Moreover, the follow-up duration was sufficient to perform a survival analysis, and > 70% of the patients died during the follow-up period, which supports the validity of the HAP-related risk scores. Finally, we focused on HAP-related risk scores, which use several clinical characteristics obtained at the time of TACE. In contrast to other risk-prediction scores, such as the STATE score, which incorporates C-reactive protein level (a test not routinely performed in clinical practice [18]), these simple-to-use risk-stratification strategies (sum of variables, each worth one point) may be of use in HCC management. In addition, sequential evaluation of HAP-related scores can be performed from the first TACE session, and the time interval between TACE sessions does not impact the results. In contrast to the Assessment for Retreatment with TACE (ART) score [19], these scores enable risk stratification in the presence of metachronous HCC nodules and in cases in which HCC treatment using TACE is insufficiently effective, irrespective of the time interval between TACE sessions.

Previous studies have shown that radiological response evaluation after TACE is a significant risk predictor in HCC patients treated with TACE [4, 18, 19]. The ART score uses radiological response based on EASL criteria measured after TACE [19]. In addition, the SNACOR model uses radiological responses based on mRECIST criteria after TACE [4, 20]. Because the ART and SNACOR models, which use post-TACE variables, were established to identify candidates who would tolerate and benefit from repeated TACE, not to identify candidates who should start TACE as the first-line treatment, these models cannot be used clinically to decide on TACE treatment initiation. Although it is also important to perform TACE to check tumor characteristics by evaluating the initial response to TACE and to re-assess the long-term prognosis accordingly, the chance of suboptimal response to the first TACE treatment still remains, which may lead to poorer prognosis, compared to pursuing other treatment modalities.

Meanwhile, because HAP-related risk scores do not include variables involved in treatment response and in changes of liver function after TACE, they can be used starting from the first TACE session. Thus, in a recent study that proposed a sequential algorithm for selecting optimal candidates for repeated TACE [21], HAP score was best suited for screening patients prior to initial TACE, whereas sequential use of ART score improved early detection of TACE failure. However, the 90-day interval criterion, which is the basis of establishing ART scores, was not adopted in this HAP-ART sequential model. Furthermore, the combination of mHAP and mHAP-II scores, instead of the conventional HAP score, was not tested. Thus, further studies are warranted to compare the predictive accuracy of different combination strategies using other models, such as the SNACOR model without a time limitation between TACE sessions [20], as well as mHAP [14] or mHAP-II [15].

Comparison of prognostic accuracy among HAP-related risk scores has been considered controversial. While several studies have validated their prognostic accuracy [14, 22], a comparison with mHAP-II scores was not available in these studies. In other studies, the prognostic accuracy of mHAP-II score was significantly better than that of HAP score [15, 23]. Furthermore, the prognostic performance of mHAP score was unsatisfactory in comparison with the mHAP-II or HAP scores [15]. When considering the prognostic significance of tumor number, which is incorporated into various HCC staging systems, such as BCLC staging and that of the American Joint Committee on Cancer (AJCC) [17, 24], as well as the association of tumor multiplicity with incomplete response after the first TACE and unfavorable long-term outcome [3], the inclusion thereof in mHAP-II score may render it superior to the HAP or mHAP scores. However, further validation studies are still required.

Although a recent study showed that development of progression or need for three rounds of TACE within the first 6 months are predictive of TACE refractoriness with anticipated poor outcomes [25], early optimization of treatment strategies, even at baseline, using HAP-related risk scores or after the first TACE (ART and SNACOR) is warranted, rather than adhering to ineffective, repeated TACE treatments. Indeed, mHAP-II score differs with TACE-refractoriness in that it was established to identify potential poor responders “before TACE,” whereas the current concept of TACE-refractory was established based on treatment responses “after several rounds of TACE.” Thus, further studies are also required to identify potential poor responders by modifying the discordant strategies. If poor prognosis is anticipated, modifications of treatment options, such as trans-arterial radioembolization, combined therapy with sorafenib, or external radiotherapy, or clinical trials can be considered.

This study had several limitations. First, the alternative treatments used after unsatisfactory results of TACE during the follow-up period might have influenced overall survival. Second, because we compared the prognostic power of HAP-related risk scores, we did not analyze other models that use post-TACE variables (e.g., the ART and SNACOR models). Thus, the predictive power of combinations of pre-TACE and post-TACE variables need to be evaluated. Third, TACE was performed during the study period even in individuals with class C–D HAP-related risk scores at the first and second TACE rounds due to the non-availability of other treatment options; this might have influenced the results of the survival analysis. Finally, although we analyzed a large sample from two institutions to increase statistical reliability, inter-institutional variability, particularly with regard to the TACE technique and baseline variables, might have biased the results. To resolve this issue, a well-designed prospective study with stratification using a predefined algorithm based on mHAP-II score is needed to validate our results. In addition, the reproducibility of the predictive power of HAP-related risk scores after a third TACE session and in patients of other ethnicities needs to be investigated.

## Conclusions

In conclusion, we demonstrated the feasibility of dynamic risk assessment by sequential evaluation of HAP-related risk scores before a first and second round of TACE in patients with HCC. Among HAP-related risk scores, mHAP-II score showed superior performance for identifying patients who would benefit from single and repeated TACE sessions. In addition, we proposed an integrated algorithm including sequential evaluation of mHAP-II score to identify optimal candidates for TACE as the first-line treatment modality and to determine the benefits of subsequent TACE sessions. This algorithm may also facilitate identification of subgroups of patients at risk of early treatment failure during repeated TACE sessions. Further studies should seek to investigate the predictive performance of combinations of various, currently available risk-prediction scores for the long-term outcomes of patients with HCC undergoing repeated rounds of TACE.

## Additional files

Additional file 1: **Figure S1**. Flow diagram of the study population selection from the two institutions. After excluding 58 patients according to our exclusion criteria, 619 treatment-naïve patients with HCC who were treated with TACE were finally included in the statistical analysis. HCC, hepatocellular carcinoma; TACE, trans-arterial chemoembolization; MPV, main portal vein; BCLC, Barcelona Clinic Liver Cancer (TIF 320 kb)

Additional file 2: **Table S1** Scoring strategies of HAP-related risk scores. **Table S2**. Comparison of baseline characteristics between the two institutions. **Table S3**. Univariate Cox regression analysis to identify risk factors for mortality (DOCX 30 kb)

Additional file 3: **Figure S2**. Schematic flow of patients at the first and second TACE according to mHAP-II, mHAP, and HAP scores. TACE, trans-arterial chemoembolization; mHAP, modified hepatoma arterial-embolization prognostic (TIF 397 kb)

Additional file 4: **Figure S3**. Kaplan-Meier curve for survival in the entire study population. Until the end of the follow-up, the median survival was 30.0 (95% CI 26.8–33.2) months and the survival rate at 1-, 3-, and 5-years after the first TACE was 76.7, 44.2, and 27.3%, respectively. CI, confidence interval; TACE, trans-arterial chemoembolization (TIF 218 kb)

## Abbreviations

AFP: alpha-fetoprotein
AIC: Akaike information criteria
AJCC: American Joint Committee on Cancer
AUROC: areas under receiver-operating curve
BCLC: Barcelona Clinic Liver Cancer
CI: confidence interval
CT: computed tomography
HAP: hepatoma arterial-embolization prognostic
HBV: hepatitis B virus
HCC: hepatocellular carcinoma
HCV: hepatitis C virus
HR: hazard ratio
IQR: interquartile range
mHAP: modified HAP
mRECIST: modified Response Evaluation Criteria in Solid Tumors
MRI: magnetic resonance imaging
TACE: trans-arterial chemoembolization
