# Supplementary figures and images for: Feasibility of dynamic risk assessment for patients with repeated trans-arterial chemoembolization for hepatocellular carcinoma
Source: BMC Cancer. 2019 Apr 16;19:363. doi: 10.1186/s12885-019-5495-6 (PMC6469056; doi:10.1186/s12885-019-5495-6)

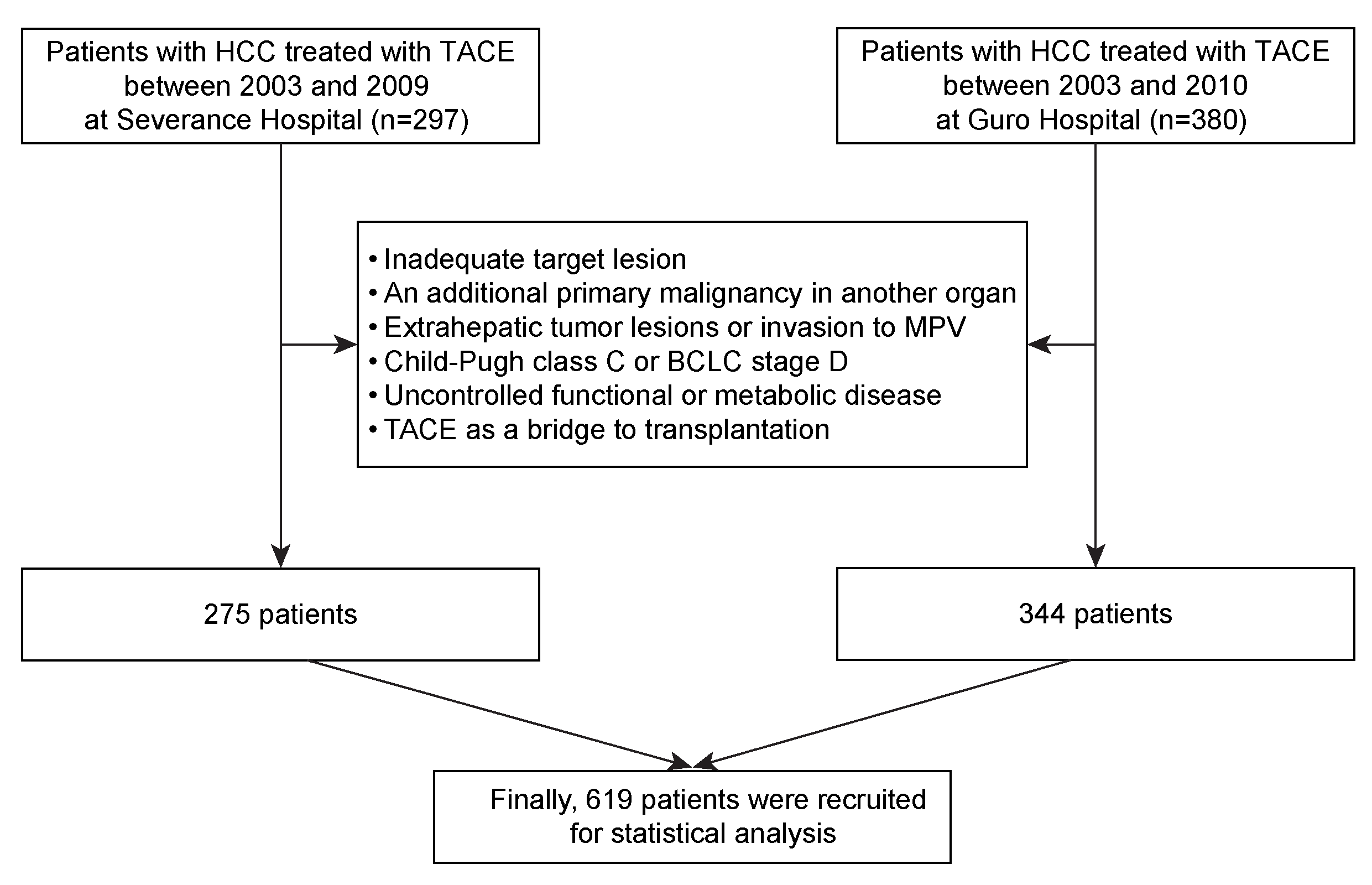

Supplement: Supplementary file 1 — Figure S1. Flow diagram of the study population selection from the two institutions. After excluding 58 patients according to our exclusion criteria, 619 treatment-naïve patients with HCC who were treated with TACE were finally included in the statistical analysis. HCC, hepatocellular carcinoma; TACE, trans-arterial chemoembolization; MPV, main portal vein; BCLC, Barcelona Clinic Liver Cancer (TIF 320 kb) [file 12885_2019_5495_MOESM1_ESM.tif]

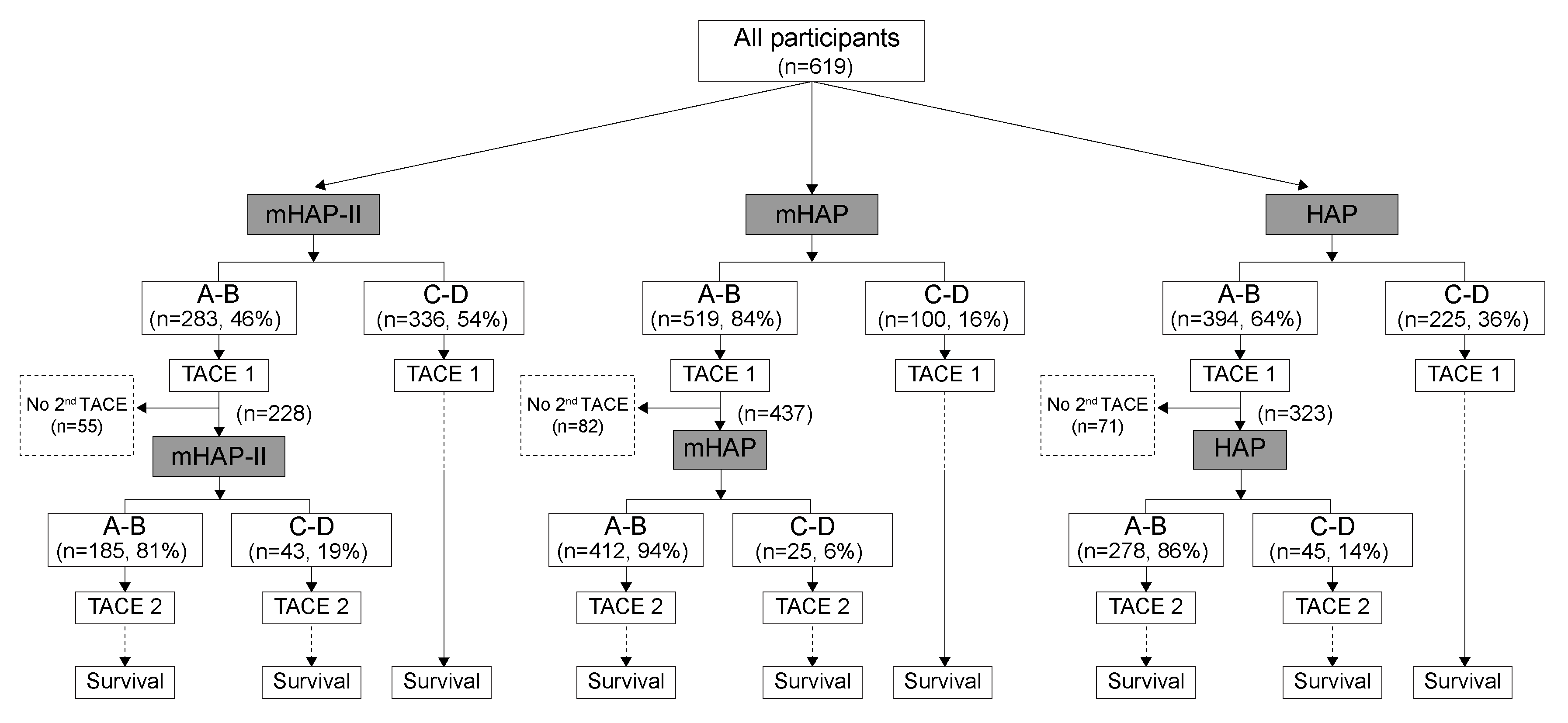

Supplement: Supplementary file 3 — Figure S2. Schematic flow of patients at the first and second TACE according to mHAP-II, mHAP, and HAP scores. TACE, trans-arterial chemoembolization; mHAP, modified hepatoma arterial-embolization prognostic (TIF 397 kb) [file 12885_2019_5495_MOESM3_ESM.tif]

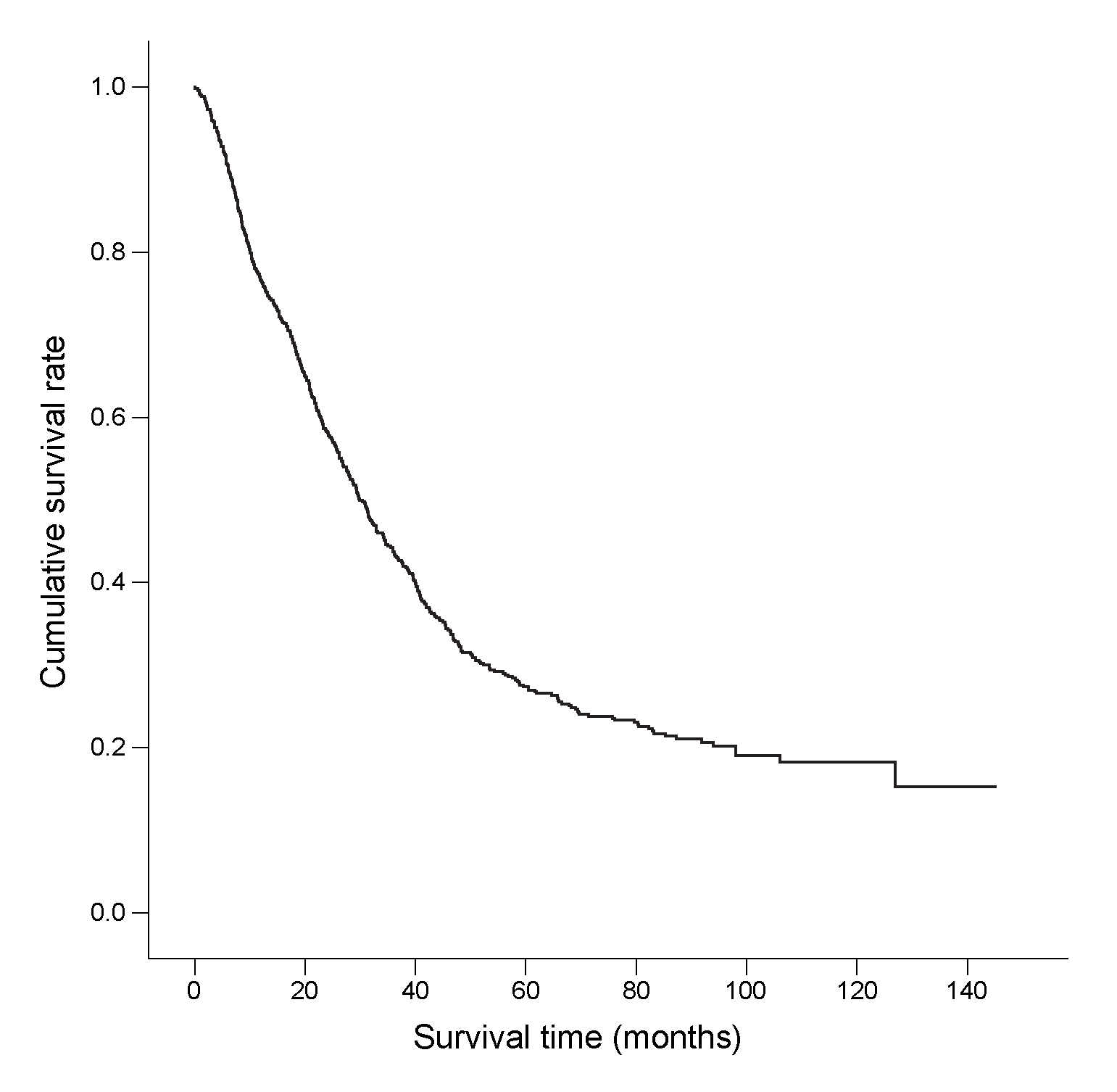

Supplement: Supplementary file 4 — Figure S3. Kaplan-Meier curve for survival in the entire study population. Until the end of the follow-up, the median survival was 30.0 (95% CI 26.8–33.2) months and the survival rate at 1-, 3-, and 5-years after the first TACE was 76.7, 44.2, and 27.3%, respectively. CI, confidence interval; TACE, trans-arterial chemoembolization (TIF 218 kb) [file 12885_2019_5495_MOESM4_ESM.tif]
