# Supplementary material for: Feasibility of dynamic risk assessment for patients with repeated trans-arterial chemoembolization for hepatocellular carcinoma
Source: BMC Cancer. 2019 Apr 16;19:363. doi: 10.1186/s12885-019-5495-6 (PMC6469056; doi:10.1186/s12885-019-5495-6)
Supplement: Supplementary file 2 — Table S1 Scoring strategies of HAP-related risk scores. Table S2. Comparison of baseline characteristics between the two institutions. Table S3. Univariate Cox regression analysis to identify risk factors for mortality (DOCX 30 kb) [file 12885_2019_5495_MOESM2_ESM.docx]

**Additional file 2: Table S1**. Scoring strategies of HAP-related risk scores

| Author | Model name | Variables and risk allocation | Point |
| --- | --- | --- | --- |
| Kadalayil [14] | HAP | Serum albumin < 36 g/dL | 1 |
|  |  | AFP > 400 ng/mL | 1 |
|  |  | Total bilirubin > 17 µmol/L | 1 |
|  |  | Tumor size > 7 cm | 1 |
|  |  | HAP A class | 0 points |
|  |  | HAP B class | 1 points |
|  |  | HAP C class | 2 points |
|  |  | HAP D class | >2 points |
| Pinato [15] | mHAP | Serum albumin < 36 g/dL | 1 |
|  |  | AFP > 400 ng/mL | 1 |
|  |  | Tumor size > 7 cm | 1 |
|  |  | mHAP A class | 0 points |
|  |  | mHAP B class | 1 points |
|  |  | mHAP C class | 2 points |
|  |  | mHAP D class | >2 points |
| Park Y [16] | mHAP-II | Serum albumin < 36 g/dL | 1 |
|  |  | AFP > 400 ng/mL | 1 |
|  |  | Total bilirubin > 17 µmol/L | 1 |
|  |  | Tumor number ≥ 2 | 1 |
|  |  | Tumor size > 7 cm | 1 |
|  |  | mHAP-II A class | 0 points |
|  |  | mHAP-II B class | 1 points |
|  |  | mHAP-II C class | 2 points |
|  |  | mHAP-II D class | >2 points |

HAP, hepatoma arterial-embolization prognostic; APF, alpha-fetoprotein.

**Table S2**. Comparison of baseline characteristics between the two institutions

| Variables | Severance Hospital (n=275) | Guro Hospital (n=344) | *P* value |
| --- | --- | --- | --- |
| Age (years) | 60 (53 - 68) | 59 (51 - 65) | 0.018 |
| Male gender | 206 (74.9) | 283 (82.3) | 0.026 |
| Etiology |  |  |  |
| HBV/ HCV/ others | 178 (64.7)/ 44 (16.0)/ 53 (19.3) | 244 (70.9)/ 46 (13.4)/ 54 (15.7) | 0.257 |
| Child-Pugh class |  |  |  |
| A/ B | 247 (89.8)/ 28 (10.2) | 269 (78.2)/ 75 (21.8) | <0.001 |
| BCLC stage |  |  |  |
| 0/ A/ B/ C | 0/ 153 (55.6)/ 100 (36.4)/ 22 (8.0) | 36 (10.5)/ 108 (31.4) / 127 (36.9)/ 73 (21.2) | <0.001 |
| Tumor size (cm) | 3.1 (2.0 - 5.1) | 3.8 (2.1 - 7.8) | <0.001 |
| Tumor number |  |  |  |
| Unifocal/ multifocal | 139 (50.5)/ 136 (49.5) | 195 (56.7)/ 149 (43.3) | 0.128 |
| Alpha-fetoprotein (ng/mL) |  |  |  |
| ≤400/ >400 | 191 (69.5)/ 84 (30.5) | 280 (81.4)/ 64 (18.6) | 0.001 |
| Segmental portal vein invasion | 22 (8.0) | 61 (17.7) | 0.002 |
| Total bilirubin (mg/dL) | 0.8 (0.5 - 1.1) | 1.0 (0.7 - 1.4) | 0.006 |
| Serum albumin (g/dL) | 4.0 (3.6 - 4.3) | 3.7 (3.3 - 4.2) | <0.001 |

Variables are expressed as median (interquartile range) or n (%).

HBV, hepatitis B virus; HCV, hepatitis C virus; BCLC, Barcelona Clinic Liver Cancer.

**Table S3.** Univariate Cox regression analysis to identify risk factors for mortality

| Variables | At the first TACE | |  | At the second TACE | |
| --- | --- | --- | --- | --- | --- |
|  | HR (95% CI) | *P* value |  | HR (95% CI) | *P* value |
| Age (years) | 0.99 (0.99 - 1.01) | 0.785 |  | 1.00 (0.99 - 1.01) | 0.594 |
| Male gender | 1.29 (1.02 - 1.62) | 0.031 |  | 1.10 (0.86 - 1.41) | 0.461 |
| Etiology |  | 0.948 |  |  | 0.973 |
| HBV | 1 |  |  | 1 |  |
| HCV | 0.97 (0.75 - 1.26) |  |  | 0.97 (0.74 - 1.28) |  |
| Others | 0.97 (0.75 - 1.24) |  |  | 0.98 (0.75 - 1.29) |  |
| Child-Pugh class |  | <0.001 |  |  | 0.006 |
| A | 1 |  |  | 1 |  |
| B | 1.56 (1.24 - 1.97) |  |  | 1.47 (1.12 - 1.93) |  |
| BCLC stage |  | <0.001 |  |  | <0.001 |
| 0 - A | 1 |  |  | 1 |  |
| B - C | 1.64 (1.36 - 1.98) |  |  | 2.54 (2.07 - 3.12) |  |
| Tumor size (cm) |  | <0.001 |  |  | <0.001 |
| ≤7 | 1 |  |  | 1 |  |
| >7 | 1.98 (1.60 - 2.45) |  |  | 2.93 (2.20 - 3.91) |  |
| Tumor number |  | <0.001 |  |  | <0.001 |
| Unifocal | 1 |  |  | 1 |  |
| Multifocal | 1.88 (1.56 - 2.27) |  |  | 1.84 (1.50 - 2.27) |  |
| Alpha-fetoprotein (ng/mL) |  | <0.001 |  |  | <0.001 |
| ≤400 | 1 |  |  | 1 |  |
| >400 | 1.62 (1.31 - 2.00) |  |  | 3.27 (2.40 - 4.45) |  |
| Total bilirubin (mg/dL) |  | 0.006 |  |  | 0.066 |
| ≤0.9 | 1 |  |  | 1 |  |
| >0.9 | 1.29 (1.10 - 1.55) |  |  | 1.22 (0.99 - 1.52) |  |
| Serum albumin (g/dL) |  | <0.001 |  |  | <0.001 |
| ≥3.6 | 1 |  |  | 1 |  |
| <3.6 | 1.51 (1.25 - 1.83) |  |  | 1.75 (1.42 - 2.15) |  |
| mHAP-II score |  | <0.001 |  |  | <0.001 |
| A-B class | 1 |  |  | 1 |  |
| C-D class | 2.07 (1.71 - 2.50) |  |  | 2.22 (1.81 - 2.73) |  |

TACE; transarterial chemoembolization; HR, hazard ratio; CI, confidence interval; HBV, hepatitis B virus; HCV, hepatitis C virus; BCLC, Barcelona Clinic Liver Cancer; HAP, hepatoma arterial-embolization prognostic.
